# Cell‐embedded microgels as emerging miniature 3D tissue‐mimics toward biochip‐based toxicity screening

**DOI:** 10.1002/btm2.70061

**Published:** 2025-09-08

**Authors:** Margaux Delafosse, Estelle Regnault, Jasmin Gebauer‐Barrett, Andreas Manz, Baeckkyoung Sung

**Affiliations:** ^1^ KIST Europe Forschungsgesellschaft mbH Saarbrücken Germany; ^2^ Ecole Supérieure de Chimie Organique et Minérale Compiègne France; ^3^ Department of Chemical & Biological Engineering University of British Columbia Vancouver British Columbia Canada; ^4^ Division of Energy & Environment Technology University of Science & Technology Daejeon Republic of Korea

**Keywords:** cell‐laden microgel, microarray chip, microphysiological system, tissue mimicry, toxicological evaluation

## Abstract

Recent developments in synthetic three‐dimensional (3D) gel microenvironments for cell culture have enabled the advancement of bioengineered organ‐specific cell niches that resemble the native 3D tissue architecture and mechanics. In particular, the application of 3D cell cultures based on miniaturized hydrogel scaffolds for toxicological analyses is attracting increasing interest because of their facile adaptability to on‐chip systems and potential as novel in vitro screening tools. We summarize the current progress in microgel‐based 3D cells integrated into biochip platforms and their utilization for the in vitro toxicity evaluation of chemicals and drug candidates. We emphasize the development of tissue‐mimicking microgel systems combined with automated gel microarray chips and organ‐on‐a‐chip devices. This review begins with the microscale hydrogel scaffolds that encapsulate mammalian cells and are used for in vitro tissue mimicry purposes. Furthermore, an overview of microgel‐based tissue modeling approaches to toxicity testing and screening is provided, along with their technical advantages in drug discovery and alternatives to animal testing.

**Translational Impact Statement**

This review article provides a full‐scale overview on the state‐of‐the‐art biochips, in which native tissue‐like miniaturized hydrogels are integrated that can support high‐throughput cytotoxicity screening and facilitate the automation of drug development procedures.

## 1 INTRODUCTION

Microchip‐based tissue mimicry is one of the most studied topics in the interface between soft matter, bioengineering, and microelectromechanical systems (MEMS).^1^, ^2^, ^3^, ^4^ Tissue‐resembling on‐chip scaffolds are not only the model tissue constructs for biomaterials research but also have been employed as screening platforms for drug development.^5^, ^6^, ^7^ For example, rendering native tissue‐like microenvironments to culture cells implemented in digital control systems could induce improved in vivo‐like cellular responses upon the addition of drug candidates, which could promote drug discovery procedures.^8^, ^9^

In pharmaceutical research, fast and effective safety evaluation for newly synthesized compounds, biologically originated substances, or nanoscale drug‐vector complexes has been strongly required to reduce cost and time for drug development.^10^, ^11^ Conventional two‐dimensional (2D) cell‐based toxicity assays have been widely accepted because (1) they have greatly reduced concern regarding species‐specific differences in metabolic pathways (compared to in vivo animal tests),^12^ and (2) cellular monolayers are fully compatible with high‐content screening (e.g., multiplexed fluorescence readouts).^13^ However, the main limitation of 2D cell culture systems lies in reflecting in vivo‐like microenvironments; thus, they do not represent the real toxicity responses in three‐dimensional (3D) tissue or organ levels.^14^

Hydrogel‐based tissue scaffolds are well‐known platforms that serve as engineered extracellular matrices (ECMs).^15^ Defined as a swollen 3D network of crosslinked polymers, a hydrogel can be designed to provide native tissue‐like microenvironments that may regulate the physiology of seeded cells. Cell–matrix interactions can be directed to specific purposes by tuning the physicochemical properties of the host hydrogels.^16^ For instance, a wide range of cell mechanics can be regulated by varying the stiffness or (visco‐)elasticity of gel matrices.^17^ Furthermore, the modification of intracellular signaling can be achieved by conjugating transmembrane receptor‐binding molecules (such as growth factors) to the polymer chains in the gel.^18^ Conversely, embedded cells may reorganize local gel structures during migration and proliferation by secreting proteolytic enzymes.^19^ Thus, hydrogels provide an interactive ECM‐like microenvironment for encapsulated cells with predefined physical and chemical characteristics.

Various 3D cell culture models have been developed based on hydrogel platforms, in which ECM‐like properties can be engineered and controlled by optimizing the types of cell lines, bioassays, and biosensors.^20^, ^21^ Using these platforms, the formation of 3D cellular constructs can be induced in a dynamic and straightforward manner. In particular, cell‐encapsulated microscale hydrogels (i.e., cell‐laden microgels) have been extensively investigated over the last two decades owing to their rapid response to external stimuli and high adaptability to biochips and 3D bioprinting.^22^, ^23^, ^24^, ^25^

Lab‐on‐a‐chip is defined as a MEMS‐based microchip device in which multiple laboratory functions are integrated typically using microfluidic circuits and/or sensors.^2^ The lab‐on‐a‐chip formats have been extensively utilized for developing miniaturized cell/tissue culture systems in biomedical sciences, and such small‐size artificial bio‐systems are called biochips. The biochips can be implemented as static or dynamic culture models, where the former is based on standard cell culture conditions and the latter exploits continuous media perfusion on‐chip. When a biochip employs dynamic culture mechanisms to recapitulate organ‐level functions, the biochip is referred to as an organ‐on‐a‐chip. Organoid‐on‐a‐chip is a special type of organ‐on‐a‐chip system in which organoids are adopted as the 3D cell components. Table 1 summarizes the chip‐related terminologies.

**TABLE 1:** Characteristics of lab‐on‐a‐chip, biochip, cell microarray, organ‐on‐a‐chip, and organoid‐on‐a‐chip devices.

Lab‐on‐a‐chip (LOC)			
Physical LOC	Biochip (2D/3D cell‐based LOC)		
Physical and chemical analyses and sensing Complex microchannel or microchamber geometries available Flow control using miniaturized valves, actuators or micro‐pumps High‐precision manipulation, mixing, reaction, and separation of droplets Microscale impedance measurement and electrochemiluminescence detection Depending on the device type, some acellular LOCs can be classified as biochips (e.g., LOCs employing DNA/RNA or enzymes)	Static culture	Dynamic culture [microphysiological system]	
	Cell microarray	Organ‐on‐a‐chip (OoC)	Organoid‐on‐a‐chip
	Tissue‐level biomimicry 2D or 3D cell organizations in patterned arrays No microfluidics Fabricated using substrate micro‐/nanopatterning or bio‐printing Combined with high‐content (fluorescence) imaging instruments	Organ‐level biomimicry 2D or 3D cell organizations Blood flow‐mimicking microfluidics Microscale sensors integrated Inter‐ or multi‐organ crosstalk implementable	A subtype of OoC Self‐organized 3D cells Blood flow‐mimicking microfluidics Microscale sensors integrated Inter‐ or multi‐organ crosstalk implementable

High‐throughput toxicity screening using miniaturized hydrogels is one of the main applications of hydrogel‐based 3D cell assays.^26^, ^27^ Given the 3D cellular microenvironments, cells seeded in a gel can be readily applied to organ‐specific toxicity analyses. In this review, we summarize the engineering principles and current progress in creating hydrogel‐based 3D tissue mimics with biochip platforms, designed for in vitro toxicology screening. The technological background and bioindustrial implications will be explained considering such applications. Special emphasis has been placed on microgel platforms that facilitate laboratory automation and effective screening. In particular, future directions for digitalized biochip‐based assays are discussed.

## 2 CELL‐EMBEDDED MICROGEL AS AN ARTIFICIAL 3D TISSUE MODEL

### 2.1 Microgels: Micro‐engineered hydrogels for tissue scaffolding

In biomedical research, microgels usually refer to microscale hydrogel structures that are typically colloidal in aqueous solutions (or immobilized at interfaces) and range from sub‐micron to hundreds of micrometers in size.^28^, ^29^, ^30^, ^31^, ^32^, ^33^ Inside the microgel, hydrophilic polymer chains are physically or chemically crosslinked to form a microscale 3D matrix. Such microgel matrices are used in a swollen state in cell culture media as elastic polymer networks that are highly permeable to ions, small molecules, and low‐molecular‐weight macromolecules. When microgel matrices are designed to encapsulate cells, the matrix polymer type is selected to be suitable for the physiology of the target cells to support cellular functions.^34^, ^35^, ^36^ For example, if adherent cells are to be embedded, network‐forming polymers should provide cell‐binding motifs, such as the arginine‐glycine‐aspartate sequence.^37^ When trapping non‐adherent or spheroid‐forming cells in a microgel matrix, the chemical requirements for gel networks are generally simpler.^38^, ^39^ Engineered microgel matrices support cell viability, migration, proliferation, and/or differentiation in response to matrix elasticity and concentration gradients of oxygen or signaling factors.^40^ Briefly, embedment of cells in a microgel is designed to produce an in vivo‐like pericellular matrix, which promotes cellular physiology and metabolism resulting in engineered 3D microtissue.^36^, ^41^ Compared to conventional 2D culture systems, which lack physiological tissue structures, cell embedment within a microgel is advantageous for studying complex cell–ECM interactions that regulate cell fate. In addition, the microgel typically offers a physically optimal platform (owing to its optical transparency) that is highly compatible with live cell imaging techniques,^40^ which is usually not available from large‐size hydrogel scaffolds. There are important advantages of encapsulating cells within microgels in comparison to the bulk hydrogel‐based cell embedment methods^22^: (1) Direct integration of cell‐embedded microgels into a lab‐on‐a‐chip system can be easily achieved. (2) Individualized high‐precision cell culture is possible using single cell‐laden microgel units. (3) Hierarchical assembly of heterogeneous microgels (encapsulating multiple cell types in a single cell‐level) can be exploited for the bottom‐up reconstruction of complex tissue interfaces.

### 2.2 Fabrication techniques for encapsulating cells in microgels

Tailoring the cellular microenvironment often requires sophisticated methods because of the highly sensitive and complex responses of cellular physiology to ambient factors. To minimize the damage to cells during encapsulation, gel fabrication protocols should be carefully designed according to their biological relevance. Various methods have been demonstrated to efficiently trap a wide range of cell types in microgel matrices with a controlled cell number, gel shape/size, polymer concentration, and mesh size of the polymer network.^29^, ^42^ Encapsulation strategies^41^ have exploited engineering methods for mini‐ and micro‐emulsification,^43^ droplet microfluidics,^26^, ^44^, ^45^ jet‐based microfabrication,^46^ electro‐spraying techniques,^47^ stop‐flow lithography,^48^ and bioprinting‐associated lab‐on‐a‐chip techniques.^49^ Subsequent crosslinking has been conducted to minimize the harmful effects on the incorporated cells and simultaneously ensure gel stability during 3D culture procedures.^26^, ^50^ Such mild crosslinking methods include gelation through local temperature control (“thermal crosslinking”), ion‐mediated crosslinking of charged polymers (“ionic crosslinking”), and ultraviolet (UV) light‐induced crosslinking (“photo‐crosslinking”).^51^ For example, Mohamed et al.^52^ developed an integrated microfluidic device that could continuously produce cell‐laden gel microspheres. The device enabled fully controlled droplet manipulation and efficient encapsulation of murine fibroblasts (NIH/3T3 cell line) in monodisperse gelatin methacrylate (GelMA) microgels, crosslinked on‐chip with UV irradiation. The synthesized microgels were shown to be an optimal platform for 3D cell culture in vitro with varying cell seeding densities. A deliberate cell‐biological design is required for the entire fabrication process to maintain maximum cytocompatibility because, in most cases, the cells are mixed with the gel precursor solution (and sometimes crosslinking agents with initiators) from the beginning.^26^ The final size and shape of the microgels are predominantly determined according to the interfacial properties of the precursor droplets, architecture of the biochip channels and chambers, and/or photomask pattern design.^53^

### 2.3 Versatile tissue mimicry using microgel platforms

**FIGURE 1:**
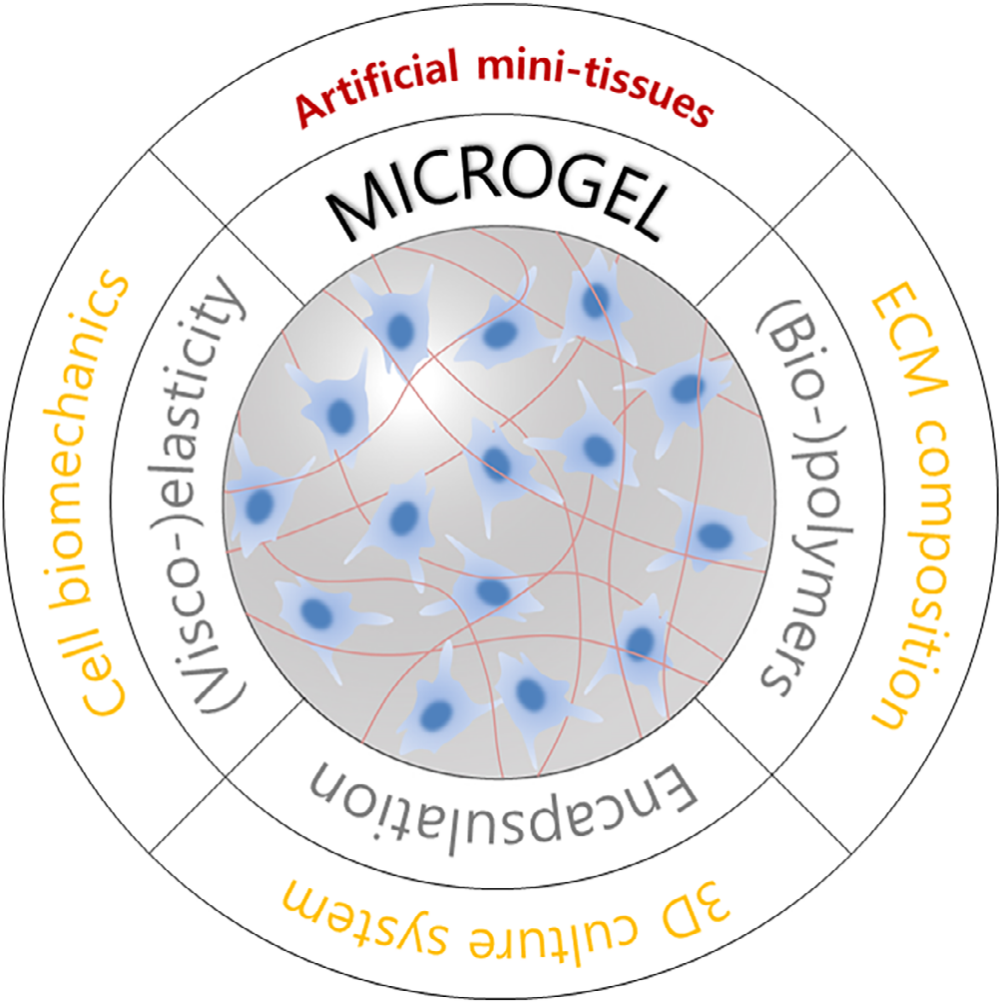
Microgel as an artificially reproduced and miniaturized tissue construct. Physical and chemical properties of gel matrices can be directly related to the main features of in vitro microtissues. For example, the elasticity (or viscoelasticity), type of (bio‐)polymers, and encapsulation capability of a microgel determine the biomechanics of interacting cells, reconstituted ECM composition, and 3D culture efficiency, respectively.

Microgels have prominent advantages in 3D cell culture that can promote the creation of miniaturized artificial tissue constructs (Figure 1). In addition to tissue‐like 3D supramolecular structures with hydrophilicity, microgels may provide facilitated diffusion and exchange of oxygen, nutrients, and metabolites (in the case of low polymer concentrations) which are required to maintain cellular vitality.^25^, ^26^, ^38^ This is enabled by the high porosity of the gel matrices and the high surface‐to‐volume ratio of the microgels. The biomechanical characteristics of a microscale 3D network that are compatible with those of the target tissues can be adjusted by varying the polymer concentration and/or crosslinking degree.^54^ Additionally, biochemical cues can be introduced by engineering the polymer components of the hydrogels.^55^ Tunable biodegradability can be rendered in microgel matrices depending on cellular properties and specific applications.^50^, ^56^ Microgels can be adapted for use as either culture media‐suspended particles with multiple morphologies (spherical, cubic, discoidal, rod‐like, and lock‐and‐key shapes)^34^, ^57^ or as 2D/3D micropatterned substrates^58^ that are compatible with organs‐on‐a‐chip concepts. Specifically, cell‐laden microgels are optimal building blocks for the bottom‐up assembly of spatially organized tissue complexes.^59^ The tissue mimicry is implemented by interfacial effects or patterning‐based self‐assembly methods.^60^ This has also been considered a 3D‐tissue assembly strategy used for organoid‐based tissue engineering applications.^61^ Such modular features of microgels enable the engineering of heterogeneous tissue structures comprising diverse populations of independently formed microgels that encapsulate various cell types and/or bioactive factors.^34^ Depending on the design of microgel building blocks, tunable microarchitectures and predefined compositional properties can enhance the microphysiological functionalities of complex 3D tissue constructs.^62^

### 2.4 Cell‐laden microgels for application to 3D in vitro assays

The prominent biomimetic capabilities of microgel matrices enable their application in versatile cell‐based in vitro assays in drug discovery, gene therapy, and tissue engineering.^63^, ^64^, ^65^, ^66^ These are typically coupled with high‐content imaging and high‐speed fluorescence microscopy. Under predefined intercellular distances and physical confinement in a microgel, 3D cells respond more reliably to external stimuli. Using cell‐laden microgel platforms, various kinds of bioassays can be applied and performed at the scale of multiple and single cells owing to the high spatiotemporal controllability of cell–cell and cell–matrix interactions. The creation of 3D co‐culture systems is also possible when different cell lineages are co‐encapsulated in a microgel.^40^, ^67^, ^68^ Compared to traditional 3D culture methods, such as spinner flasks, rotary bioreactors, and bulk hydrogels, microscale in vitro assay systems require drastically lower amounts of cells and reagents, and screen a wider range of libraries of chemicals in shorter time scales.^69^ Table 2 shows the 3D cell culture characteristics of bulk hydrogels, microgel platforms, and organoid models for the comparison in the context of in vitro toxicology.

**TABLE 2:** Comparison between bulk hydrogel‐, microgel‐, and organoid‐based in vitro 3D cell toxicity testing models.

	Bulk hydrogel‐based models	Microgel‐based models	Organoid models
Formation of 3D‐cell structures	Cell seeding in the ECM‐mimicking gel precursors followed by physical or chemical crosslinking (with minimized cytotoxic effects)		Spontaneous cellular organizations to form native organ‐like small‐size architectures
ECM–cell interactions	Relatively strong		Relatively weak
Cell‐to‐cell interactions	Relatively weak		Relatively strong
Extent of in vivo mimicry	Relatively low		Relatively high
Biophysical and biochemical cues of the matrices	Highly tunable		Less tunable than gel‐based models
Diffusive transport of oxygen, nutrients, and wastes in the 3D‐cell structures	Limited by low surface‐to‐volume (S/V) ratio. However, high water content in the gels may enhance the permeability when the polymer concentration is low	Highly permeable due to both high S/V ratio and high water content (when the polymer concentration is low)	Compact arrangement and high density of cells may hinder molecular diffusion, particularly when the organoid size increases
High‐content (fluorescence imaging‐based) screening	Less compatible	More compatible	
Feasibility (reproducibility) of in vitro assay outcomes	Relatively high		Relatively low
Relevance to organ‐specific endpoints	Relatively low		Relatively high

## 3 MICROGELS AS 3D TISSUE MIMICS FOR THE USE IN TOXICITY SCREENING

### 3.1 3D cellular microenvironments and organ‐specific toxicity analyses

One of the main reasons for drug development failure in preclinical trials is the inadequacy of drug toxicity evaluation, partly because of the large experimental data gap between in vitro and in vivo tests. Current in vitro 2D cell platforms are mostly based on static mono‐ or bilayer culture formats that usually lack physiological relevance. Thus, the utilization of either 3D cell‐laden microgels or microarray/microfluidic biochips must be highlighted to recapitulate in vivo‐like microenvironments. Microfluidic‐combined and/or automated biochip systems can also be used to model the dynamic aspects of cellular responses upon exposure to drug candidates.

Macroscopic and microscopic hydrogel scaffolds are the most popular biomaterials used to implement 3D cell culture systems.^70^, ^71^, ^72^, ^73^ Cells can be pre‐seeded in gel matrices or induced to form multilayers of different cell types as a co‐culture platform on a hydrogel surface. Cellular proliferation and interactions can be controlled by tailoring the chemical composition and biophysical features of the polymer networks.

Organ‐specific niches in cell‐laden hydrogels promote physiologically relevant in vitro toxicology evaluations that are similar to in vivo models, especially when empowered by 3D bioprinting and microfabrication technologies.^56^, ^74^, ^75^, ^76^ Drug candidates in pharmaceutical research have been screened through various tests, including enzymatic reaction kinetics and solubility properties in a therapeutic formulation. The drug screening can be efficiently performed using droplet microfluidics for diverse chemical libraries.^77^, ^78^ Spatial and temporal control over multicellular organizations and dynamics is implemented in engineered microphysiological systems, where multiplexed biomaterials often play a central role.

### 3.2 Microgel‐based 3D cell microarray chips

**FIGURE 2:**
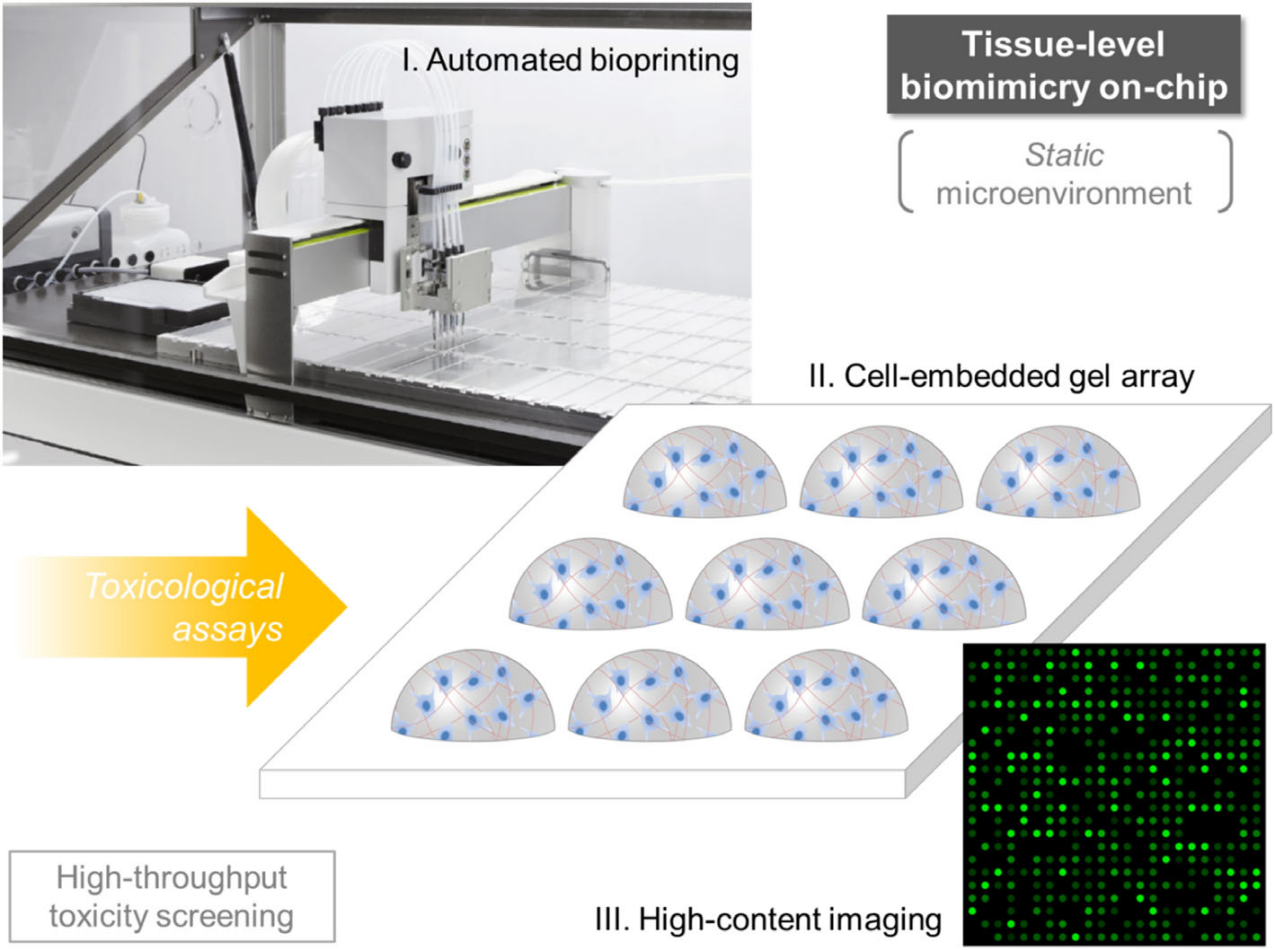
Microarray of gel spots used for cytotoxicity screening. This platform provides static microenvironments for the 3D cells embedded in microgels, enabling tissue‐level biomimicry on‐chip (Table 1). Such biochips are typically fabricated through automated bioprinting of cell‐laden gel arrays. They are then treated with in vitro toxicological assays to be finally screened through high‐content imaging. Upper left image: A digitally controlled microarray printer [Credit: GeSiM mbH]. Lower right image (microarray chip) by Thomas Shafee, CC BY 4.0 license.^94^

Microgel‐based platforms are optimal for automated drug screening methods (Figure 2) due to the microgels' high compatibility to robotic microcontact printing techniques and intrinsic adaptability to high‐throughput assessment systems.^27^ Microgel nano‐/biotechnology is an emerging field for the implementation of miniaturized assays to screen libraries of toxic materials and drug molecules.^56^, ^79^, ^80^, ^81^, ^82^ Robotic microspotting can be used to create microarrays of cell‐trapped microgels on functional substrates,^83^, ^84^ which enable high‐throughput toxicity tests. Microgels can also be used as bioink materials for automated 3D printing.^85^, ^86^, ^87^, ^88^ Cell‐laden microgels can be integrated in another bioink solution or scaffold for printing.^34^, ^89^, ^90^ A digital microfluidic method has been developed to create arrays of cell‐laden microgels with controllable shape and content.^91^ In general, 3D cell microarray chips do not provide the features of dynamic cell culture based on media microflows, but are highly adapted to laboratory automation frameworks.^92^, ^93^

The most important microscale gel‐based method for high‐throughput drug (or microenvironmental factor) screening involves the fabrication of microchips (or mini‐chips) in which cell‐embedded microgel arrays are spotted.^12^, ^95^, ^96^, ^97^ These 3D cellular microarray platforms can be utilized to generate high‐volume in vitro screening data by emulating the structural and functional characteristics of native target organs, such as the liver, bone, and neural systems. This type of platform operates on the basis of digitally controlled bioprinting (microspotters) and/or high‐content advanced microscopy systems, which are highly compatible with an automated laboratory concept.^98^ Using tissue‐mimicking microgel arrays, on‐chip acquisition of dose–response profiles can be implemented between multiple combinations of drug candidates and cellular metabolic markers (Table 3).

**TABLE 3:** 3D cell‐embedded gel array microchips for high‐throughput toxicity screening.

Device configuration	Chip fabrication	Microgel array integration with cells on‐chip	Cell type and culture period	Mode of toxicity testing and screening	Test compounds and doses	References
Complementary coupling of a micropillar chip and a microwell chip → Stamping of the two chips enables the cells in the former contact the culture medium in the latter	Plastic injection molding‐based manufacturing of the micropillar chip (532 pillars with 0.75 mm in diameter) and the microwell chip (532 wells with 1.2 mm in diameter)	Cell‐laden ECM gel spots on the micropillars by covalent attachment of ECM polymers onto the pillar surface (spotting 60 nL of cell suspension in Matrigel onto the Matrigel‐coated pillar followed by gelation)	Human liver epithelial cell line, THLE‐2 (1‐day pre‐incubation, 1‐day incubation with viruses, and 2‐day incubation with test compounds on‐chip)	Fluorescence imaging‐based whole chip scanning for screening 84 combinations of human metabolic gene expressions (for Phase I enzymes like CYPs and Phase II enzymes like UGTs)	Acetaminophen, bromfenac, flutamide, tamoxifen, trifluoperazine, troglitazone (evaluated with a dose‐dependent manner, 0.01–10,000 μM)	Kwon et al.^99^
Miniaturized array of spatially addressable pattern of 3D cell spots on a functionalized glass slide	Gel microarray printing on a glass slide pretreated with 3‐(aminopropyl)trimethoxysilane, and then treated with poly(sterene‐*co*‐maleic anhydride) (PS‐MA)	Cell‐seeded type I collagen or alginate microgels (50 μm‐thick) patterned on a PS‐MA‐treated slide using a microarray spotter	Human breast cancer cell line, MCF7 (5 days' culture)	Fluorescence imaging‐based microarray scanning for assessing cell viability and CYP‐generated metabolites	Doxorubicin, 5‐fluorouracil, tamoxifen with dose–response cytotoxicity profiles (10–10^6^ nM)	Lee et al.^100^
Digitally sculpted gel arrays with spatially heterogeneous microgel structures	Multilayer photo‐lithography and high‐precision alignment on a glass substrate	Photo‐lithographically cross‐linked microgels containing cells and ECM components using multiple masks with microscale control (microgel anchoring at specified coordinates in 3D space)	Primary neurons, embryonic stem cells, human umbilical vein endothelial cells, and fibroblasts	N/A	N/A	Gurkan et al.^101^
Microgel array on a surface‐functionalized slide glass	Automatic printing of gel microarray on a glass slide functionalized with 3‐(trimethoxysilyl)propyl methacrylate	Cell‐laden gel array platform (400 spots) fabricated with a robotic microarray spotter	Bone‐marrow derived human mesenchymal stem cell (hMSC)	Evaluation of osteogenic cell differentiation by analyzing the expression of alkaline phosphatase using automated high‐content imaging (not directly related to the toxicity testing)	N/A	Dolatshahi‐Pirouz et al.^102^
3D cellular microarray platform printed on a pre‐patterned slide glass	Dry‐patterning of the mixture of poly‐l‐lysine and Ba^2+^ onto poly(styrene‐*co*‐maleic anhydride) coated glass slide, followed by gel microarray spotting	Spotting of cell‐alginate mixture (30–60 nL/spot) in a 6 × 8 × 8 array and physical gelation in situ	Human hepatoma cell line, HepG2 (24 h drug incubation and subsequent 72 h culture)	Evaluation of antiproliferative effects and protein expression profiles involved in proliferation, angiogenesis, adhesion, and drug metabolism (CYP enzymes, β1‐integrin, vascular endothelial growth factor, etc.)	Doxorubicin, tamoxifen, amitriptyline, and 5‐fluorouracil	Meli et al.^103^
			Immortalized human neural stem cell line, ReNcell VM (6 days' culture)	High‐throughput dose–response assessment of cell viability and differential toxicity	(i) Developmental neurotoxicants, such as cadmium chloride (0.1–100 μM), retinoic acid (0.1–100 μM), and dexamethasone, (1–1000 μM) and (ii) antiproliterative anti‐cancer agent, 5‐fluorouracil (0.1–100 μM)	Meli et al.^104^

For instance, Lee et al.^100^ and Kwon et al.^99^ developed miniaturized 3D cell chips that enabled the rapid screening of drug compounds and metabolites. The chips comprised arrays of hemispherical (concave‐down) microgels immobilized on polymer‐functionalized glass substrates or plastic micropillar arrays. Hydrogel matrices of collagen type I, alginate, and Matrigel were used to encapsulate human mammary epithelial cells (MCF7) and liver epithelial cells (THLE‐2). Using such microarray chips, efficient large‐scale screening can be performed to obtain data on the combined expression of drug‐metabolizing enzymes (cytochrome P450) in the 3D cell spots upon exposure to model drug compounds.

Meli et al.^103^ reported a different type of microarray in which liver‐like microenvironments were reconstituted with a focus on 3D hepatocyte signaling biomarkers and the metabolic activities of xenobiotics. For chip fabrication, human hepatoma (HepG2) cell‐embedded alginate microgel spots were printed on a polymer‐functionalized glass substratum using a microarray spotter. The 3D on‐chip cell proliferation and toxicity profiles were evaluated and quantified in the presence of various doses of the drug compounds.

In other experiments, cell‐laden gel microarray platforms have been utilized to screen stem cell differentiation capacity and cytotoxicity in various 3D microenvironments and toxicant concentrations. Similar to Meli et al.,^103^ the same research group used an alginate gel microarray to evaluate the 3D culture conditions for human neural stem cells (ReNcell VM cell line) and the adverse effects of neurotoxicants on the 3D cells.^104^ Dolatshahi‐Pirouz et al.^102^ tested combinatorial 3D microenvironments for osteogenic differentiation of human mesenchymal stem cells in UV‐crosslinked GelMA microgel arrays.

From a different viewpoint, Gurkan et al.^101^ developed a fabrication method for spatially heterogeneous microgel structures in which multiple cell types (primary neurons, embryonic stem cells, human umbilical vein endothelial cells, and fibroblasts) were encapsulated to form miniaturized complex tissue architectures. This high‐precision patterning technique could be adapted for on‐chip 3D cytotoxicity screening.

### 3.3 Microgel‐integrated microfluidic chips for standard 3D cell models

**FIGURE 3:**
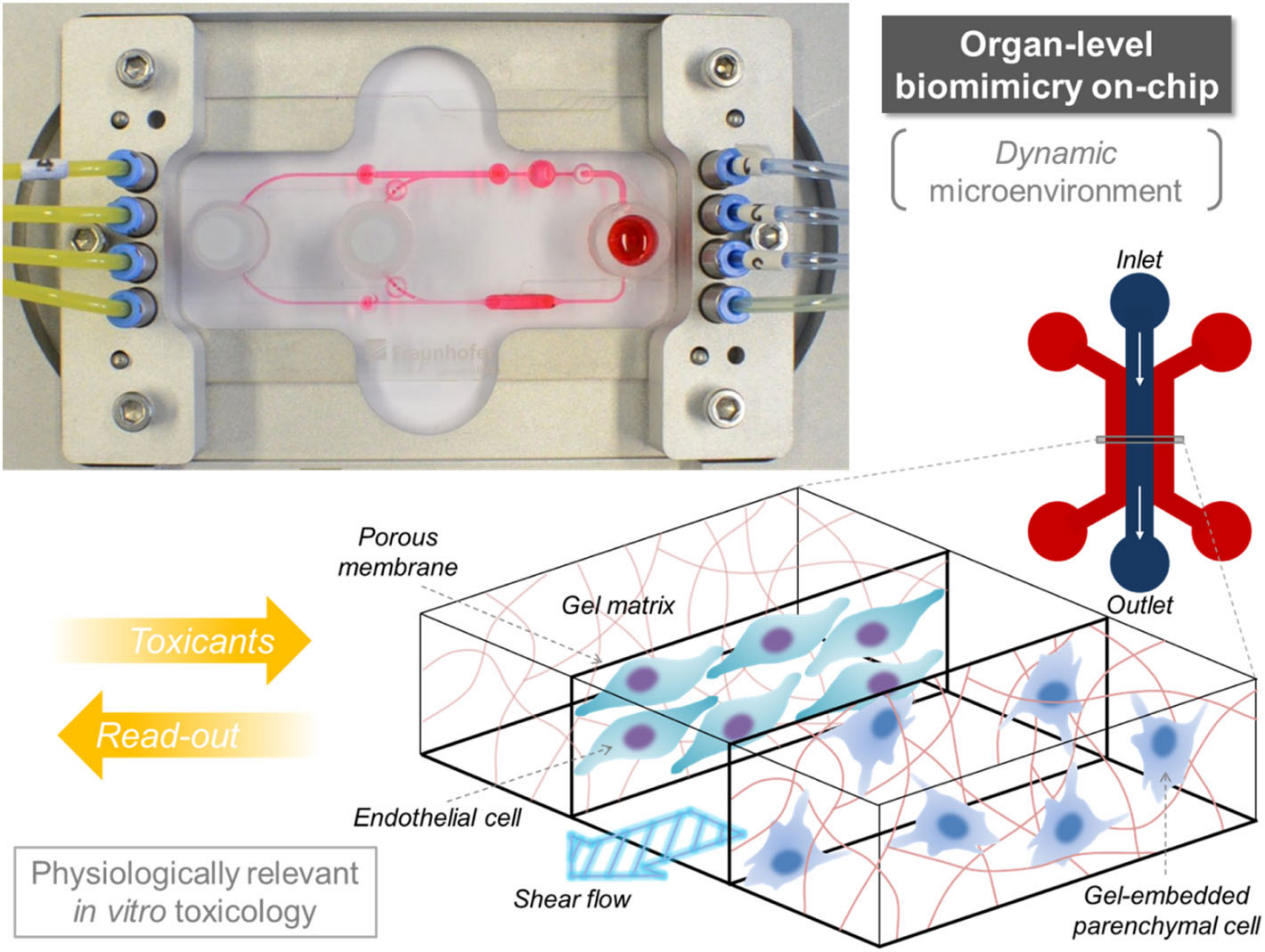
Microgel‐based organ‐level biomimicry on‐chip (Table 1). This platform exploits gel‐integrated microfluidic devices that provide dynamic microenvironments for miniaturized 3D (as well as 2D) cell culture systems. Using the organ‐on‐a‐chip tools, drug candidates or other test compounds can be delivered to the cells in an in vivo‐like manner (via blood flow‐mimicking microflows). The subsequent ex‐ or on‐chip sensing of toxicity readout implements physiologically relevant in vitro toxicology studies. Upper left image: An example of a multi‐organ chip^124^, ^125^ [Credit: Fraunhofer IWS]. Lower right image: Adapted and modified from Sung^126^ with permission by Taylor & Francis Group.

Microfluidic techniques are of significant importance for the implementation of in vivo‐like tissue mimicry, in which biofluids dynamically affect cell/tissue physiology through shear flow, physical actuations, and/or diffusion–reaction processes.^105^, ^106^, ^107^, ^108^, ^109^, ^110^, ^111^ Miniaturized tissue constructs, implemented as a compartment on a microfluidic device, can be designed in various shapes and dimensions to be under the influence of perfusion at a controlled flow rate to mimic blood‐tissue interactions. Tissue‐like microgels (or gel‐based microtissues) can be readily deployed in organ‐on‐a‐chip systems.^112^, ^113^, ^114^, ^115^, ^116^ The on‐chip generation and manipulation of individual microgels can be achieved using typical microfluidic channel configurations and associated fabrication methods.^117^, ^118^, ^119^ This approach has two main advantages compared to the traditional 2D culture platform (Figure 3). First, instead of applying test drugs or chemicals directly on the 2D cell layer, they may be delivered through blood circulation‐like perfusion techniques to the model 3D tissue to better simulate in vivo situations.^120^, ^121^ Next, the responses of cells to the effects of drugs, especially toxic effects, can be significantly better reflected in a 3D system, which is close to the hierarchical tissue architecture of the living body.^122^, ^123^ Furthermore, tunable concentration gradients of chemicals, morphogens, and growth factors can readily be formed in microfluidic channels to precisely induce cellular responses. A summary on the microphysiological systems for toxicological assessments is provided in Table 4.

**TABLE 4:** Gel‐integrated microfluidic biochips developed for toxicological evaluations of 3D cells in vitro.

Device configuration	Channel architecture	Microgel integration with cells on‐chip	Cell type and culture period	Automation and/or parallelization	Mode of toxicity testing and screening	Test compounds and doses	References
Integration of a 3D culture chamber device and a multiplex fluid controller	3D culture platform comprising 200 individual chambers (610 μm in height), and coupled with an overlaying layer of channel (455 μm in height)	Each chamber containing cell aggregate or organoid‐embedded gels (Matrigel)	3D aggregates of a cancer cell line (MDA‐MB‐231) and patient‐derived pancreatic and colon organoids [on‐chip growth for over 14 days]	Channel array divided into 20 subsets of 10 individual chamber units accommodate 10 different patient samples and enable 20 independent fluidic conditions	Quantification of cell death and apoptosis under: (i) continuous exposure of individual drugs for 72 h or pulse‐like exposure for a 4 h drug/s, and (ii) dynamic exposure of combination of drugs in pulses	Clinically relevant doses of chemotherapeutic drugs such as gemcitabine (100 nM), paclitaxel (10 nM), 5‐fluorouracil (100 nM), oxaliplatin (100 nM), and cisplatin (100 nM)	Schuster et al.^127^
Digital microfluidics platform that is based on the electrodynamic manipulation of single/multiple droplet(s)	Device assembled with an electrodes‐patterned bottom plate and an unpatterned top late joined by spacers of ~180 μm thick (a droplet retention barrier implemented in each culture region)	Droplets of collagen‐cell suspensions loaded in the device and allowed to gel	Co‐culture of HepG2 and NIH‐3T3 cell lines in a collagen type I‐based microgel, forming a liver organoid (4 days' culture)	Droplets manipulated on‐chip for merging and splitting by applying sinusoidal potential to bottom‐plate electrodes suing an automated high‐voltage switching system	On‐chip liver organoid contractility and cell viability assessments after 24‐h exposure to the test compound, followed by off‐chip analyses on albumin concentration and enzymatic activity of cytochrome P450 (CYP)	Non‐opioid analgesic and antipyretic agent, *N*‐acetyl‐*para*‐aminophenol (acetaminophen; 0, 5, 10, and 20 mM)	Au et al.^128^
A PDMS microchamber for tissue culture was implemented in which polycarbonate membranes formed the top and bottom surfaces	Continuous perfusion of culture medium through the microchamber membranes (from down to top)	Liver slices imbedded in a hydrogel (Matrigel; 10 μL) to prevent attachment to the microchamber membrane	Precision‐cut liver slices of the rats (3 days' culture on‐chip)	N/A	(i) Tissue viability assessment by measuring the enzyme, lactate dehydrogenase, leaked from injured hepatocytes, (ii) phase I and phase II (CYP‐mediated) metabolisms determined using 7‐ethoxycoumarin and 7‐hydroxycoumarin, respectively	N/A	Van Midwoud et al.^129^
Assembled platform that incorporates a bioprinted dot array of 3D cell constructs in the bioreactor	Multilayer bioreactor structure with the inlet (bottom) and outlet (top) fluidic channels (total volume of the system: 2.4 mL)	Photocrosslinkable GelMA microgels containing hepatic spheroids grown on‐chip with continuous perfusion of media	Spheroids formed from the human liver cell line HepG2/C3A (30 days' culture on‐chip)	On‐chip microgel array fabrication using a bioprinter based on robotically controlled dual bio‐print heads (one for placing cells and the other for depositing a gel matrix)	(i) Monitoring of the secretion rates of albumin, alpha‐1 antitrypsin, transferrin, and ceruloplasmin, (ii) immunostaining for the hepatocyte markers, cytokeratin 18, MRP2 bile canalicular protein and tight junction protein ZO‐1	*N*‐acetyl‐*para*‐aminophenol (15 mM)	Bhise et al.^130^
Bioprinted tubule‐like lumen in a gel‐filled microchamber	3D convoluted renal proximal tubule on‐chip through which controlled perfusion of media is available	3D open lumen embedded in a gelatin‐fibrinogen gel matrix in a microchamber (where an epithelial layer covers the inner surface of lumen)	Human immortalized proximal tubule epithelial cell (PTEC; 6 weeks' culture)	On‐chip printing of the lumen structure (with fugitive ink) using a motion‐controlled 3D bioprinter	Gene expression analysis, cytokine analysis, albumin uptake study, and diffusional permeability measurements (for assessing epithelial barrier disruption)	Nephrotoxin, cyclosporine A (0, 10, 50, and 100 μM)	Homan et al.^131^
A body‐on‐a‐chip comprising interconnected 3 chambers in which cell‐laden gels are formed on polycarbonate membranes	The channel layout connects inlet and outlet reservoirs through 3 chambers for organ‐mimics.	Cells and cell aggregates pre‐mixed with type I collagen solution: Pipetting into the organ chamber followed by collagen gel polymerization	Spheroids formed from human colon cancer cell line HCT‐116, human liver cell line HepG2/C3A, human leukemia cell line HL‐60 (culture <1 week)	Pumpless and gravity‐driven unidirectional perfusion of culture media through the organ chambers and interconnecting channels	Cell viability test and spheroid size analysis	Tegafur (200/800 μM regimen)	LaValley et al.^132^
A skin chip comprising 2 PDMS layers separated by a porous membrane, supported by gravity‐driven flows	Dermis and epidermis equivalents constructed on the PDMS layers: Fluidic channels are formed underneath the porous membrane.	Fibroblasts‐embedded collagen gel compartment on‐chip: Keratinocytes cultured on the hydrogel	Primary dermal fibroblasts and keratinocytes (<1 week), human vascular endothelial cells (HUVEC‐2; <1 week), human vascular endothelial cells (HUVEC‐2), leukocytes (HL‐60 cell line)	Pumpless media perfusion by tilting the chip with a gravity‐flow device	Assessments of the viability of fibroblasts (doxorubicin) or the extent of immune cell recruitment (UV irradiation)	Doxorubicin (<36 μM) or UV irradiation	Kwak et al.^133^
OrganoPlate® 2‐lane (Mimetas): 96 two channel glass and plastic microfluidic chips	Two adjacent channels: Organ (liver) channel and perfusion channel	Injection of hepatocytes‐suspended collagen type I solution into the inlet of the organ channel	Differentiated iPSC iCell 2.0 hepatocytes, differentiated THP‐1 cells, HMEC‐1 endothelial cells (on‐chip culture up to 15 days)	Automated liquid handling system	Measurements of cell viability, albumin production, and lactate dehydrogenase (LDH) activity	Troglitazone (180 μM, 72 h treatment)	Bircsak et al.^134^
Wafer‐based silicon chip including integrated electrodes and a microcavity	Microcavity implemented for the 2D cells on a transparent membrane formed between two gold electrodes in the microchannels	N/A	Human alveolar basal epithelial cell line A549, human liver cell line HepG2, human renal proximal tubule epithelial cell line Th‐1	Parallel operation of the microfluidic cartridge with electrodes integrated in a miniature incubator microscope	Bio‐assays for cell viability and DNA damage; Impedance measurement for barrier integrity	Cisplatin (20 μg/mL, 24 h exposure) or silica nanoparticles (100 μg/mL, 24 h exposure)	Kohl et al.^135^
Digital microfluidic device bearing patterned chromium electrodes that enables single droplet manipulation	Valve‐less fluid manipulation and organoid‐droplet exchange procedure controlled by programmed electrical signals	On‐chip controlled droplet exchange between cell‐laden collagen and reagent solutions	Organoids formed from human liver cell line HepG2 with/without murine fibroblast cell line NIH‐3T3 (4 days culture on‐chip)	Fully automated and miniaturized system for droplet manipulation, organoid encapsulation, and micro‐tissue formation	Assays for cell viability and contractility, albumin secretion, enzymatic activity (cytochrome P450 3A4), and drug‐induced hepatotoxicity	Acetaminophen (0, 5, 10, and 20 mM)	Au et al.^128^
A microfluidic chip incorporating 29 microwells connected through a single fluid channel	Each well filled with microgel where organoids are embedded: Drug perfusion through the connecting channel	Cell‐suspended Matrigel solution introduced into the microwell‐channel structure → Chip micro‐centrifugation and channel flushing → Gelation	Patient‐derived lung cancer organoids (3 days culture on‐chip)	Single‐step microgel embedment and formation of multiple organoids	Cell viability assay, apoptosis analysis, and organoid size measurement	Cisplatin and etoposide (48 h treatment)	Jung et al.^136^
A programmable membrane‐valve‐based microfluidic chip (multiplexer control device)	Matrigel‐filled multiplex microchambers where organoids are embedded: Multiple drug treatments through the connecting channels	Organoid‐suspended Matrigel solution injected into the microwell‐channel structure → Organoid settlement followed by media perfusion	Patient‐derived pancreatic tumor organoids and colon organoids, aggregates of cancer cell line MDA‐MB‐231 (7 days culture)	Fully parallelized high‐throughput drug screening with multiple conditions	Analysis of cellular death and apoptosis	Drug combination of CPT‐11 (100 nM), oxaliplatin (100 nM), fluorouracil (100 nM), gemcitabine (100 nM), and paclitaxel (10 nM) [72 h treatment]	Schuster et al.^127^

Using a different approach, a bottom‐up strategy has been adopted to integrate on‐chip 3D liver mimics based on self‐aggregated hepatocytes.^130^ In this study, hepatic spheroids (generated by the HepG2/C3A cell line) spontaneously formed in microwells were mixed with a GelMA solution and printed as droplets in a microfluidic culture chamber. The GelMA droplets were immediately crosslinked by UV light illumination to form liver spheroid‐encapsulated microgel spots on the bottom of the chamber, and a long‐term (30 days) dynamic 3D culture on‐chip was demonstrated.

The ultimate goal of these technological approaches is to create in vitro biochips in which all native tissue‐mimicking elements are integrated into a microfluidics‐supported and digitally automated device.^137^, ^138^, ^139^, ^140^, ^141^ In such devices, each organ‐representing 3D tissue compartment is connected through microfluidic channels that implement physiological functions such as adsorption–distribution–metabolism–excretion (ADME). These multi‐organ chips can be easily coupled with programmed light microscopy platforms to enable high‐image‐content‐based evaluation systems that can be used for high‐throughput screening of the toxic effects of drugs or other chemicals.

### 3.4 Microgel‐embedded organoid‐based biochips

Organoids are robust models for spontaneously organized 3D cell assemblies, which are formed as “mini‐organs” in artificially regulated in vitro microenvironments, and closely resemble their in vivo counterparts.^142^, ^143^, ^144^ The bottom‐up self‐organization of cells into a 3D mini‐organ is critically different from the top‐down fabrication methods of organ‐on‐a‐chip devices or microgel‐array biochips.^145^, ^146^, ^147^, ^148^, ^149^, ^150^, ^151^ Miniaturized hydrogel matrices have been proven to provide optimal ECM‐like features for the pattern formation and morphogenesis of embedded organoids, where modular ligand composition, local stiffness, viscoelasticity, porosity, and matrix degradability could be finely controlled.^152^, ^153^, ^154^, ^155^ Therefore, hydrogel scaffold‐based organoid models have been extensively developed to overcome the drawbacks of conventionally cultured organoids, such as inefficient production, uncontrolled size, and morphological and functional heterogeneity.^156^ Encapsulating organoids in microgels (or microgel assemblies) may be advantageous because they can be produced with high monodispersity at a relatively high yield using conventional microfluidic setups. This is advantageous for facilitating screening, standardization, and automation.^157^, ^158^ The generation of a large number of cell‐entrapped microgels enables high‐throughput screening of a wide range of combinatorial chemical conditions.^40^ By varying the libraries of cell lines and 3D microenvironments, extensive screening of a given set of chemicals can be efficiently performed for preclinical efficacy and/or toxicity testing in the pharmaceutical and cosmetics industries.

Furthermore, the microgel platforms can be readily adapted for combined toxicity testing. Given the narrow size distribution, replicas of the cell‐laden microgel batches can be prepared to evaluate several combinations of chemical substances in a single screening process. The toxicity and effects of chemicals on cellular fate can be examined in terms of biophysical and biochemical cues, with properties identical to those of microgel matrices.^26^ Directed assemblies of cell‐laden microgels enable in vitro drug testing by using higher‐order complexes of 3D tissue mimics.^56^ The drug development cycle can be accelerated using these miniaturized screening systems. In addition, a combination of tissue‐like microgels and microfluidic chips can be used for both environmental sampling and digitalized biosensing.

Miniaturized gel matrices have been extensively developed to support directed self‐organization of stem cells into organoids on microfluidic chips.^152^ Droplet microfluidics has been widely used for the high‐throughput production of organoid microgel capsules.^159^ Using a microfluidic electrospraying technique, murine primary cell‐based gastrointestinal organoids were encapsulated in the core‐shell structure of Matrigel and alginate matrices for cryopreservation.^160^ In addition, an automated biofabrication and culture system was developed to generate uniform Matrigel microspheres, in which human tumor‐derived organoid precursors were seeded.^161^ Each organoid‐laden microgel was then placed in a microplate well, and genetic and metabolic analyses were conducted for further application in high‐throughput drug screening and personalized medicine.

Various microfluidic devices with/without bioprinting have been developed as microgel‐based organoid‐on‐a‐chip platforms to support drug discovery pipelines in the pharmaceutical industry.^162^, ^163^ For example, a microfluidic culture device has been developed to test the efficacy of chemotherapeutic drugs in patient‐derived lung cancer organoids.^136^ In this platform, each cell‐laden Matrigel matrix was filled in a well of a 29‐microwell chip and grown to be a single organoid per well under a dynamic culture condition, which was used for evaluating the test drug's ability to induce cell apoptosis in a dose‐dependent manner. A similar concept was adopted in a different microfluidic device design, where combinatorial drug efficacy was evaluated on human tumor organoids embedded in Matrigel‐filled microchambers. The anti‐tumor effect (i.e., cytotoxicity) was examined by quantifying cell death and apoptosis under dynamic exposure conditions of chemotherapeutic drugs.^127^

### 3.5 Other biochips incorporating small‐scale hydrogels

Several types of biochip devices were developed for in vitro cytotoxicity testing by incorporating small‐size hydrogel constructs, compartments, and templates (rather than using typical microgel beads or fillings) in combination with microfluidic platforms. For example, Van Midwoud et al.^129^ reported a microfluidic culture system in which liver slice‐embedded hydrogel compartments were integrated. This top‐down gel embedding method prevented the attachment of liver slices to the chamber wall and supported prolonged cell viability and metabolic activity.

A more complex 3D tissue architecture was fabricated using a bioprinting technique based on a hydrogel‐filled microchamber platform.^131^ A gelatin‐fibrinogen gel‐based convoluted lumen architecture was implemented in a microfluidic device to capitulate the renal proximal tubule. The 3D lumen structure was lined with human immortalized proximal tubule epithelial cells (PTECs), and the controlled perfusion of culture media through the lumen resulted in enhanced differentiation and polarization of the PTEC phenotype and associated functionalities. Using the miniaturized gel‐based 3D cell system, the adverse effects of a nephrotoxic compound (cyclosporine A) on the reconstituted epithelium were tested to further investigate the mechanisms of drug‐induced kidney injury.

Pumpless microphysiological systems have also been developed to harbor miniaturized hydrogel‐based tissue compartments. Pumpless microfluidic chips have significant advantages in terms of their structural simplicity and operational expediency for wet‐lab end users. LaValley et al.^132^ invented a unique design for an organs‐on‐a‐chip device, whose biological functionality was maintained by the gravity‐driven unidirectional flow of culture media. In this platform, multiple human tissue constructs such as colon cancer spheroids (HCT‐116 cell line), hepatocytes (HepG2/C3A cell line), and promyoblasts (HL‐60 cell line) were embedded in collagen gel compartments, which were interconnected by blood vessel‐mimicking fluid channels. The utility of the system was demonstrated by a cytotoxicity test using a chemotherapeutic drug (tegafur).

Kwak et al.^133^ reconstructed an on‐chip human dermis by encapsulating primary dermal fibroblasts and keratinocytes in collagen gel matrices. The skin chip was operated by the gravity‐driven flow of culture media through channels lined with human vascular endothelial cells (HUVEC‐2 cell line), where leukocytes (HL‐60 cell line) were allowed to circulate. This platform was utilized to evaluate anticancer drug toxicity upon exposure to doxorubicin, as well as to test the immune responses to ultraviolet irradiation.

Bircsak et al.^134^ seeded 3D cell aggregates in an ECM‐like gel component on a commercially available chip‐based toxicity screening system to recapitulate in vivo liver function. The liver chip incorporated an endothelial layer‐lined fluid channel in which macrophages were introduced to circulate in the culture media. This system demonstrated the capability for automated drug hepatotoxicity screening.

In addition, an integrated silicon microchip system for label‐free cytotoxicity assessment has been developed by interconnecting the modules of miniaturized optical microscopes and electrical impedance sensors.^135^ In each microfluidic module, a micro‐cavity was implemented for the 2D culture of human cell lines (A549 lung, HepG2 liver, or Th‐1 kidney) on a transparent membrane formed between two gold electrodes in the microchannels. Using a sensor‐based dynamic cell culture system, the adverse effects of inorganic nanoparticles were efficiently evaluated by monitoring the cell morphology and 2D layer integrity, which have the potential to be applied for gel‐based 3D cell toxicity screening.

A digital microfluidic platform was devised for on‐chip manipulation of individual droplets of a mixture of cell suspensions and collagen type I solution.^128^ This platform enabled the on‐chip formation of gel components and subsequent self‐organization of hepatic organoids (based on HepG2 and NIH‐3T3 cell lines) in the gel matrix. Its capability as a model of drug toxicity evaluation was demonstrated by metabolizing nervous system drugs in vitro.

Interestingly, the integration of organoid‐embedded gel components on a chip enabled a fully automated system in which interconnected multi‐organ cultures with real‐time biosensing were implemented.^164^ On this platform, liver and cardiac organoids were formed by seeding human primary hepatocytes and human induced pluripotent stem cell‐derived cardiomyocytes, respectively, in UV photo‐crosslinked GelMA (+fibrin) hydrogels, which were filled in biomicroreactor chambers. During photo‐crosslinking, the micropatterns were transferred to the gel matrices through photomasks, which enabled the on‐chip microfabrication of a hepatic lobule‐like pattern and a parallel‐aligned structure of cardiomyocytes. These gel micropatterns promoted the generation of native tissue‐mimicking organoid functionalities. Based on the biochip platform, organoid toxicities of the model drugs were tested in two different microphysiological systems; that is, acetaminophen toxicity for heart‐liver‐on‐chips and doxorubicin toxicity for heart‐liver‐cancer‐on‐chips, while monitoring biophysical and biochemical factors in vitro in a continuous manner.

## 4 TRANSLATIONAL IMPACT

The on‐chip platforms of microgel‐based 3D cell systems have significant implications in view of clinical and regulatory translations. First, the utilization of 3D cell culture models can facilitate the extrapolation of toxicity evaluation data between the 2D cell‐based screening and clinical trials.^12^, ^165^ Furthermore, patient‐derived cells or organoids encapsulated in ECM‐like 3D microgels on‐chip can be a robust in vitro testing tool for promoting precision medicine, which supports personalized toxicity predictions by avoiding the potential of patient‐to‐patient variation. In addition, physiologically based toxicokinetics of drug compounds can be directly evaluated on‐chip when in vivo‐like microenvironments and inter‐organ cross‐talks are reconstituted by adding microfluidic media circulation to the 3D cell systems. This enables efficient ADME modeling and drug safety evaluations using biochips in the early stage of preclinical research. The biochip‐generated toxicity datasets can supplement the in vitro*–*in vivo knowledge gap and may support the 3R principle (replacement, reduction, and refinement) of animal testing in preclinical safety studies.

Similarly, the 3D‐cell biochips can be utilized to improve the safety predictions of environmental chemicals and drug compounds. In a tight relationship with the 3Rs, the biochip systems may serve to elaborate the concept of quantitative adverse outcome pathways (qAOPs), which were conceived by the Organization for Economic Co‐operation and Development (OECD).^166^, ^167^ Within the qAOP framework, predictive models in mechanism‐based toxicology can be easily exploited for regulatory gap filling. Main regulatory bodies, including the U.S. Food and Drug Administration (FDA) and the European Medicines Agency (EMA), are actively engaged in the translational research initiatives of microfluidic biochips.^12^, ^168^ Progress in standardization and regulatory applications of biochips may additionally accelerate commercial manufacturing of the systems in the medical device industry. Commercially available microphysiological systems used for the risk assessment of chemicals are extensively reviewed by Nitsche et al.^169^ The emerging clinical translation examples include (i) microfluidic platforms for skin mimicry, (ii) microfluidic intestinal models, (iii) dynamic culture‐based liver mimics, and (iv) multi‐organ chips for reconstructing skin–liver, skin–liver–heart, intestine–liver, and intestine–liver–brain–kidney connections.^169^

## 5 LIMITATIONS AND CHALLENGES

Despite multiple promising characteristics of the microgel‐integrated biochips for toxicity screening, there still remain technical bottlenecks that hamper large‐scale applications of the devices in clinical and industrial stages. Regarding the biological components, human cell lines are the most widely adopted cell source for in vitro toxicological evaluations. Yet the cell lines frequently overexpress toxicity‐relevant proteins, which may limit high‐precision toxicological tests. Primary cells are particularly advantageous for personalized in vitro modeling, but are only suitable for acute toxicity testing owing to their short lifespan in culture conditions. The use of stem cells (and their aggregates) can be a promising alternative to overcome such limitations, although the differentiation protocols of stem cells are usually time‐consuming and restricted by wide variability in efficiency and robustness.^171^ In addition, when cells are encapsulated in biopolymer microgels, the gel matrices may undergo biodegradation (e.g., in a few days for Matrigel) or be consumed by cells rapidly, so that the gel compartments become unstable over time during the culture period. Microgels comprising synthetic biocompatible polymers are superior in terms of degradation‐resistant properties, although the synthetic gel matrices generally exhibit lower ECM relevance when compared to the biopolymer microgel materials.

To enable in vivo‐like microenvironments for embedded cells, the miniaturized artificial ECM gels should closely recapitulate the microstructures and physical characteristics of native tissues. Nevertheless, the ECM‐mimicking biomaterials used in biochips are commonly based on simple homogeneous and isotropic hydrogel matrices. Since most organs develop in asymmetric milieux in terms of geometry and mechanics, fine structurization of microgel matrices may be needed to recapitulate anisotropic or patterned (sub)micron‐scale architectures in cardiac and nerve tissues in vivo. This may be achieved, for instance, by exploiting biomimetic liquid crystal technologies.^170^ Formation of the local gradient of tensile strain^173^ or the self‐assembled scaffolds of rod‐shaped microgels^66^ could be effective ways to create structured ECM‐like gel matrices. Multifaceted microgels also can be a potential option for organizing asymmetric matrices.^22^ This direction of research is still in its infancy, and biochip‐oriented methods specifically need to be developed.

The 3D ECM gel microarray chips straightforwardly enable high‐throughput cytotoxicity assessment and combinatorial screening of compounds.^174^, ^175^ Nevertheless, they mostly rely on static culture conditions so that neither flow‐driven cues (i.e., shear stress) nor temporal control of cellular microenvironments can be provided. Furthermore, standard microarray formats may not support compartmentalized medium chambers for individual gel matrices, which allows unwanted cellular communications (between different gel spots) through the common medium.^176^ In contrast, the 3D cell‐embedded microfluidic chips, including organoid‐on‐a‐chip platforms, have robustness in handling biological fluid dynamics and chemical exposure of the microphysiological systems. However, these devices typically require complex hardware set‐ups and operational procedures, which often result in convoluted kinetics and low throughput.^166^, ^171^ The complication in device operation also requires additional time/cost for end‐user training.

The lack of standardization and validation capability is the major limitation for the toxicological translations of all types of microgel‐integrated biochips. To overcome this barrier, the fabrication methods and protocols should be transparent and transferable either in device/software engineering or in cell biological techniques to underpin the reproducibility and interpretation of toxicity readouts.^166^ The 3D‐cell biochip systems should allow quick decision making and need to be validated with large‐scale toxicity datasets of reference compounds.^172^ For clinically relevant validation, the use of individual patients' stem‐cell‐derived cells will be more desirable than relying on the immortalized human cell lines, which have been widely adopted for proof‐of‐concept device developments.^121^ Coupling with in silico prediction toolkits based on machine learning technologies may support the development of powerful validation processes. In the domain of biochips, well‐defined validation protocols are still largely missing, and close collaborations between different stakeholders (e.g., regulatory agencies and pharmaceutical industries) are strongly required.

## 6 CONCLUSIONS AND PERSPECTIVES

Over the last two decades, cell encapsulation techniques using microgels have rapidly emerged and have been highlighted as new methodologies for fabricating 3D tissue mimics. A wide range of tissue types can be regenerated using cell‐embedded microgel systems under either short‐term or long‐term culture conditions. With progress in the microengineering technology of soft materials, more sophisticated microgel models have been applied to pharmaceutical technologies and biomedical engineering. In light of this, microgel‐based 3D cell toxicity assays have been recently illuminated as a new frontier of such applications. As microgel technology is closely related to microfluidics, there is a natural connection between cell‐encapsulated microgels and lab‐on‐a‐chip platforms. In a microfluidic device, the production of cell‐embedded microgel components, formation of 3D tissue constructs, interconnected micro‐perfusion, and transport of toxicants and metabolites, automated imaging, and toxicity analyses can be implemented in a single integrated system. In this system, the direct application of physiologically based toxicokinetics is possible, which can be modeled in higher complexity using the concept of ADME‐toxicity (ADMET). For 3D‐cell microarray chips, high‐content imaging and high‐speed image analysis technologies should be further developed to support increased throughput data processing, which may be assisted by emerging machine learning tools. Ultimately, biochip‐based tissue‐like microgels are expected to reduce the reliance of bio‐industry sectors on animal experimentation.

## CONFLICT OF INTEREST STATEMENT

The authors declare no conflicts of interest.

## Data Availability

Data sharing is not applicable to this article as no new data were created or analyzed in this study.
